# Effects of Adult Male Circumcision on Premature Ejaculation: Results from a Prospective Study in China

**DOI:** 10.1155/2015/417846

**Published:** 2015-01-28

**Authors:** Jingjing Gao, Chuan Xu, Jingjing Zhang, Chaozhao Liang, Puyu Su, Zhen Peng, Kai Shi, Dongdong Tang, Pan Gao, Zhaoxiang Lu, Jishuang Liu, Lei Xia, Jiajia Yang, Zongyao Hao, Jun Zhou, Xiansheng Zhang

**Affiliations:** ^1^Department of Urology, The First Affiliated Hospital of Anhui Medical University, Hefei, Anhui 230032, China; ^2^Department of Radiology, The First Affiliated Hospital of Anhui Medical University, Hefei, Anhui 230032, China; ^3^Academy of Public Health, The Anhui Medical University, Hefei, Anhui 230032, China; ^4^Academy of Public Health, The Xiangya School of Medicine, The Central South University, Changsha, Hunan 410000, China; ^5^Department of Urology, The Chaohu Affiliated Hospital of Anhui Medical University, Chaohu, Anhui 238000, China; ^6^Department of Urology, The Anqing Affiliated Hospital of Anhui Medical University, Anqing, Anhui 246000, China; ^7^Department of Urology, The Huaibei Affiliated Hospital of Anhui Medical University, Huaibei, Anhui 235000, China

## Abstract

The purpose of this study is to investigate the effects of adult male circumcision on premature ejaculation (PE). Therefore, between December 2009 and March 2014, a total of 575 circumcised men and 623 uncircumcised men (control group) were evaluated. Detailed evaluations (including circumcision and control groups) on PE were conducted before circumcision and at the 3-, 6-, 9-, and 12-month follow-up visits after circumcision. Self-estimated intravaginal ejaculatory latency time (IELT), Patient-Reported Outcome measures, and 5-item version of the International Index of Erectile Function were used to measure the ejaculatory and erectile function for all subjects. The results showed that, during the one-year follow-up, men after circumcision experienced higher IELT and better scores of control over ejaculation, satisfaction with sexual intercourse, and severity of PE than men before circumcision (*P* < 0.001 for all). Similarly, when compared with the control group, the circumcised men reported significantly improved IELT, control over ejaculation, and satisfaction with sexual intercourse (*P* < 0.001 for all). These findings suggested that circumcision might have positive effects on IELT, ejaculatory control, sexual satisfaction, and PE severity. In addition, circumcision was significantly associated with the development of PE.

## 1. Introduction

Male circumcision is one of the most commonly performed surgical procedures in the world [1]. It consists of the surgical removal of some or all of the foreskin (or prepuce) from the penis. Approximately one-third of males in the world have been circumcised for religious, cultural, and medical reasons, as well as personal preference and several other reasons [2]. Specific benefits of male circumcision included providing substantial protection against the acquisition of HIV and preventing urinary tract infections, the transmission of some sexually transmitted infections, and penile cancer [3–5].

Although male circumcision was considered to have important benefits for male reproductive health, results from previous studies on the relationship between circumcision and sexual function were controversial. According to the survey reported by Frisch et al. [6], circumcised Danish men were more likely to report frequent orgasm difficulties (adjusted odds ratios (OR) = 3.26; 95% confidence interval (CI) = 1.42–7.47) and sexual difficulties (including sexual function difficulties (adjusted OR = 1.29; 95% CI = 0.66–2.53), premature ejaculation (PE) (adjusted OR = 1.23; 95% CI = 0.58–2.60), or erectile difficulties (adjusted OR = 1.29; 95% CI = 0.66–2.53)) than uncircumcised men.

However, another survey conducted by Bronselaer et al. [7] showed that circumcision might negatively affect male sexual status. For the glans penis, circumcised men reported decreased sexual pleasure (dorsal: 3.31% versus 3.72%, *P* < 0.001; lateral: 3.31 versus 3.57, *P* < 0.001; ventral: 3.70 versus 3.85, *P* < 0.017) and lower orgasm intensity (dorsal: 3.13% versus 3.37%, *P* = 0.006; lateral: 3.14 versus 3.31, *P* = 0.020; ventral: 3.45 versus 3.55, *P* > 0.05) than uncircumcised men. A higher percentage of the circumcised men experienced unusual sensations (e.g., burning, pricking, and itching). Additionally, for the penile shaft, the circumcised men also described a higher percentage of discomfort and pain (dorsal: 1.17% versus 1.05%, *P* < 0.001; lateral: 1.17 versus 1.05, *P* < 0.001; ventral: 1.22 versus 1.06, *P* < 0.001). A higher percentage of circumcised men reported numbness and unusual sensations at the dorsal, lateral, and ventral sides.

To the best of our knowledge, there were few studies [8] to evaluate the relationships between adult circumcision and PE in the Chinese population. Therefore, we conducted an observational and prospective study and aimed to investigate whether there are any effects of circumcision on PE in adult males in China.

## 2. Subjects and Methods

### 2.1. Subjects

Anhui is a province of China with more than 68 million inhabitants. Between December 2009 and March 2014, an interventional and prospective field survey was conducted in the five cities (namely, Huaibei city, Wangjiang city, Hefei city, Anqing city, and Chaohu city) of Anhui province, China. These cities were selected randomly to represent the northern, southern, middle, western, and eastern parts of Anhui province.

A total of 575 males who underwent voluntary circumcision for medical reasons (e.g., phimosis, recurrent balanitis) were enrolled from Andrology Outpatient Clinics in the above five cities, which represented the male population of Anhui province in terms of population distribution across geographic regions, educational and occupational status, and age groups. In addition, another 623 male volunteers with indications for circumcision who delayed the procedure by 1 year were recruited from the health examination centers in the same five cities as the control group. In order to ensure the accuracy and comparability of the survey, there was no difference between the circumcision and control groups with respect to the demographic information.

For inclusion, men had to be uncircumcised, had to be aged ≥18 years, and have had a heterosexual, stable, and monogamous sexual relationship with the same partner for at least 6 months. In addition, because some subjective questions were asked in our study, eligible men should be able to comprehend and speak Chinese.

Exclusion criteria mainly included foreskin covering less than half of the glans, a bleeding disorder, keloid formation, and other conditions that might unduly increase the risks of elective surgery. In addition, men who have had medication that could have affected their ejaculatory function were excluded (e.g., selective serotonin reuptake inhibitors and phosphodiesterase type inhibitors).

### 2.2. Study Design and Procedure

This study was reviewed and approved by the Anhui Medical University Research Subject Review Board. Before the survey, all subjects were informed about the procedure of the survey, and those who participated were asked to provide written consent.

This survey was designed as a three-stage protocol. First, before circumcision, all subjects (including circumcision and control groups) were asked to undergo a physical examination by an experienced clinician and then completed a verbal questionnaire, which included information regarding basic demographic variables (e.g., age and educational and occupational status), medical and sexual histories (e.g., self-estimated IELT, duration of the relationship, and frequency of sexual intercourse), and self-estimated scales (e.g., the Chinese version of International Index of Erectile Function-5 (IIEF-) 5 and the Patient-Reported Outcome (PRO) measures) [9–11]. The PRO measure has been used to assess PE and measure different aspects of the disorder, including perception of ejaculatory control, satisfaction with sexual intercourse, severity of PE, personal distress, and interpersonal difficulty [12–15].

In addition, based on the PE definition suggested by ISSM [16–18], men were diagnosed with lifelong PE if they experienced vaginal penetration for less than one minute, a loss of control, and/or negative sexual consequences. The IIEF-5 questionnaire contained five questions that were answered according to symptoms (scale range 0–5). IIEF-5 scores ≥22 points were rated as normal erectile function, while scores <22 were rated as erectile dysfunction (ED). The Chinese version of IIEF-5 and PRO measures have been used in Chinese male population in previous studies [15, 19, 20]. The Cronbach's alpha coefficients for the Chinese version of IIEF-5 and PRO measure in our survey were 0.79 and 0.83, which indicated acceptable internal consistent reliability.

In the second stage, men in the circumcision group received a circumcision. Under local anesthesia, the foreskin was separated from the head of the penis with a probe to fully expose the coronal groove. Scissors were used to make an incision in the foreskin on the upper side of the penis. After making the slit, the foreskin was pulled back to expose the glans. Then, the specialists cut off the foreskin around the rim of the coronal groove and used electrocautery (electric current) to control any bleeding. The incision was closed with stitches. Follow-up visits were scheduled on postoperative days 3, 8, and 30. Surgical results, adverse events, resumption of activities of daily living, and participants' high degree of satisfaction with their surgical procedures were described. In additions, participants were counselled to refrain from sexual activity for at least 30 days after the surgery.

The third stage involved follow-up visits (at 3-, 6-, 9-, and 12-months after circumcision) for both the circumcision and control groups. At each visit, all subjects underwent a standardized medical history and physical examination. Then, information on ejaculatory and erectile function was collected by an experienced clinician using a verbal questionnaire, which mainly concentrated on sexual history, and included the Chinese assessment of IELT, PRO, measure and IIEF-5. Finally, detailed records for all subjects before circumcision and at follow-up visits were entered into the database.

### 2.3. Statistical Analysis

All statistical analyses were performed using the SPSS software (SPSS, Inc., Chicago, IL, USA, version 13.0). Descriptive statistics were used to summarize the subject's characteristics. Data on demographic information for all subjects were expressed as mean ± standard deviation (SD) or number (percentage) when appropriate. Data (including self-estimated IELT, and outcomes of PRO and IIEF-5 measures) at baseline (before circumcision) and follow-up visits for all subjects were expressed as mean ± SD. Chi-square test was used to compare categorical data, and the independent *t*-test or ANOVA was used to compare numerical data. The magnitude of the association between circumcision, PE, and ED with time was assessed with odds ratios and 95% CI. Statistical significance was defined as *P* < 0.05.

## 3. Results

### 3.1. Demographic Information

Of the 1198 patients enrolled in the study, 998 (83.31%) males completed one-year follow-ups. The mean ages for circumcision (*N* = 575) and control groups (*N* = 623) at baseline were 35.29 ± 9.81 and 36.42 ± 11.30 years, respectively. The number of follow-ups at each visit was 1,122 (circumcised group: *N* = 542; control group: *N* = 580) for 3 months, 1,076 (circumcised group: *N* = 534; control group: *N* = 542) for 6 months, 1,038 (circumcised group: *N* = 528; control group: *N* = 510) for 9 months, and 998 (circumcised group: *N* = 504; control group: *N* = 494) for 12 months. There were no differences between circumcision and control groups with respect to demographic information at each follow-up visit. In addition, based on the definition of PE and ED, the incidences of PE and ED in circumcision were 13.04% and 9.57%, whereas those in the control group were 11.88% and 9.63%. Detailed demographic information for all subjects at baseline is summarized in Table 1.

### 3.2. Outcomes of IELT, PRO Measures, and IIEF-5 at Baseline

At baseline (Tables 2 and 3), there was no significant difference between circumcision and control groups with respect to self-estimated IELT, each domain of PRO measures, and IIEF-5. The mean self-estimated IELT in circumcision and control groups was 1.58 ± 0.74 and 1.55 ± 0.78 minutes, respectively. Similarly, no significant difference was also observed between the circumcision and control groups with respect to the total and each domain score of IIEF-5. The mean total score of IIEF-5 in the circumcision and control groups was 22.05 ± 4.74 and 22.01 ± 4.92, respectively.

### 3.3. Outcomes of IELT, PRO Measures, and IIEF-5 at Each Follow-Up Visit

During the one-year follow-up visits (Tables 2 and 3), men in the circumcision group experienced dramatic increases in reported self-estimated IELT at 6-, 9-, and 12-month visits, whereas they reported lower self-estimated IELT at the 3-month visit (versusat baseline). Similarly, for each domain of the PRO measures, the circumcised men also reported higher scores of ejaculatory control, sexual satisfaction, and PE severity from the 6- to 12-month visits. In addition, for each domain of the IIEF-5, significant differences were found between the circumcision and control groups with respect to sexual satisfaction in IIEF-5. However, no significant difference between circumcision and control groups was observed with regard to personal distress and interpersonal difficulty in PRO measures and other questions in IIEF-5.

### 3.4. Associations between Circumcision, PE, and ED at 3-, 6-, 9-, and 12-Month Follow-Ups

Furthermore (Table 4), circumcision was found to be associated with PE at 3-, 6-, 9-, and 12-month follow-up (3-month follow-up: OR = 1.13, 95% CI = 1.05–1.42; 6-month follow-up: OR = 0.82, 95% CI = 0.53–0.98; 9-month follow-up: OR = 0.56, 95% CI = 0.42–0.79; 12-month follow-up: OR = 0.37, 95% CI = 0.24–0.57). However, no significant relationship was found between circumcision and ED at each follow-up visit.

## 4. Discussion

PE is a multifactorial disease. Combined with self-estimated IELT, PRO measures, and IIEF-5 questions, we were able to perform a comprehensive analysis of the relationships between adult circumcision and PE. Our findings might provide a framework for understanding the effects of circumcision on sexual dysfunction in China.

Results from our survey showed that self-estimated IELT, ejaculatory control sexual satisfaction (assessed by the subdomains of PRO measures and IIEF-5), and severity of PE were significantly associated with adult circumcision. The circumcised men reported higher self-estimated IELT and better ejaculatory control, sexual satisfaction, and PE severity than the uncircumcised men. Similarly, compared with data at baseline, the self-estimated IELT, ejaculatory control, sexual satisfaction, and PE severity were also improved in the circumcised men at the 6-month visit. However, the self-estimated IELT and ejaculatory control for circumcision at the 3-month visit were significantly worse than those at baseline. No significant difference was found between the circumcised men at baseline and 3-month visit, when regarded to the scores of sexual satisfaction and personal distress. Furthermore, the association between circumcision and PE was observed at 3-, 6-, 9-, and 12-month follow-up visits. The results confirmed the above conclusion.

In the prospective survey conducted by Alp et al. [21], they found that the participants' mean IELT (assessed by stopwatch) before and after circumcision was 104.36 ± 66.21 s and 123.56 ± 54.44 s, respectively. The PE diagnostic tool (PEDT) scores (including the five questions to assess perceived control, frequency, minimal stimulation, distress, and interpersonal difficulty) were 4.26 ± 2.91 before and 2.63 ± 1.82 after circumcision. The improvements in IELT and PEDT after circumcision were statistically significant. Similar findings were also observed in the study by Namavar and Robati [22]. Their results showed that circumcision significantly improved sexual satisfaction, frequency of intercourse, and IELT. Senol et al. concluded that circumcision might contribute to sexual satisfaction by prolonging pudendal-evoked potential latency [23]. Therefore, our results confirmed the above findings in previous studies, which suggested that circumcision might positively affect ejaculatory function.

However, the conflicting findings from other studies should also be considered. Results from the study by Krieger et al. [24] reported that adult male circumcision was not associated with sexual dysfunction, for example, an inability to ejaculate, PE, pain during intercourse, and sexual displeasure. In addition, after investigating 500 couples from five countries (Netherlands, United Kingdom, Spain, Turkey, and the United States), Waldinger et al. [25] found that the mean IELT (assessed by stopwatch) in circumcised and uncircumcised men was 6.7 minutes (range 0.7–44.1 minutes) and 6.0 minutes (range 0.5–37.4 minutes), respectively. They found that time to ejaculation was significantly less.

Interesting, for the outcomes of PRO measures at follow-up visits, the self-estimated IELT and ejaculatory control for circumcision at 3-month visits were significantly worse than those at baseline. Previous studies [26–28] have shown that penile sensitivity might be increased with cutting of the foreskin in a short period. Moreover, sexual function at 3 months after circumcision might not accurately reflect sexual function at a later period [29]. Therefore, we speculated that the dramatic decrease on IELT and ejaculatory control for circumcised men might result from penile hypersensitivity and less frequency of sexual activity in the early postoperative phase.

Additionally, the IIEF-5 measure was used to evaluate the effect of adult circumcision on erectile function. Expecting sexual satisfaction, no significant difference was found between circumcised and uncircumcised men. Masood et al. [27] found that the total mean IIEF-5 was 22.41 ± 0.94 and 21.13 ± 3.17 before and after circumcision, respectively (*P* = 0.4). Similar findings were also observed in the study by Hoschke et al. [30], which showed that ED rates were not different for the 6.7% men who were circumcised and the 93.3% who were uncircumcised. However, a survey of 22 Mexican men circumcised mostly for medical reasons reported a significant improvement in the perception of erectile function after circumcision [28]. Therefore, because of controversy surrounding the relationships between circumcision and erectile function, further studies were needed.

Several limitations of the currently study should be considered. First, previous studies have reported that adult circumcision might affect the sexual function of female partners. However, we conducted a study for men only, and further studies on the effect of adult circumcision on the female partner are needed. Second, although one-year follow-up visits were conducted in our study, longer follow-up visits (e.g., two- or five-year follow-up visits) should be performed in future studies. Third, this study was patient oriented and based only on subjective reports. Objective data such as penile biothesiometry could help assess penile sensation in the future studies. Finally, because some sensitive and personal questions on sexual history were included in our study, participants' bias should also be considered.

## 5. Conclusion

With the assessments of IELT, PRO measures, and IIEF-5, our survey systematically evaluated the effects of adult circumcision on PE in China. It provided a better framework with which to determine their relationships. During one-year follow-up visits, the circumcised men experienced higher IELT and better scores of control over ejaculation, satisfaction with sexual intercourse, and severity of PE than men before circumcision. Similarly, when compared with the control group, the circumcised men reported significant improvement on IELT, control over ejaculation, and satisfaction with sexual intercourse. PE severity in the circumcision group was also less than in the control group. Circumcision was also found to be associated with PE. Further studies on the effects of circumcision on PE are still needed.

## Figures and Tables

**Table 1:** Demographic information and sexual status for all subjects (circumcision and control groups) before circumcision.

	All	Circumcision group	Control group	*P* value
	(*N* = 1198)	(*N* = 575)	(*N* = 623)	
Age, years	35.88 ± 10.42	35.29 ± 9.81	36.42 ± 11.30	0.53
Lifestyle				
Smoking	734 (61.27)	359 (62.43)	375 (60.19)	0.43
Alcohol	542 (45.24)	272 (47.30)	270 (43.34)	0.17
Educational status				0.49
Primary school	153 (12.77)	82 (14.26)	71 (11.40)	
Middle school	204 (17.03)	99 (17.22)	105 (16.85)	
High school	436 (36.39)	205 (35.65)	231 (37.08)	
University	405 (33.81)	189 (32.87)	216 (34.67)	
Occupational status				0.62
Workers	232 (19.37)	115 (20.00)	117 (18.78)	
Drivers	167 (13.94)	76 (13.22)	91 (14.61)	
Famers	295 (24.62)	141 (24.52)	154 (24.72)	
Officials	139 (11.60)	60 (10.43)	79 (12.68)	
Other occupations	365 (30.47)	183 (31.83)	182 (29.21)	
Monthly income, RMB	1630.16 ± 257.23	1,613.27 ± 244.16	1645.74 ± 280.25	0.57
Resident				0.31
Urban	768 (64.11)	377 (65.57)	391 (62.76)	
Rural	430 (35.89)	198 (34.43)	232 (37.24)	
Duration of the relationship, years	9.70 ± 4.21	9.63 ± 4.03	9.75 ± 4.59	0.41
Frequency of sexual intercourse, time/4 weeks	6.61 ± 3.24	6.45 ± 3.85	6.74 ± 4.45	0.39
Self-estimated IELT, minutes	1.56 ± 0.75	1.58 ± 0.74	1.55 ± 0.78	0.74
IIEF-5, scores	22.03 ± 4.82	22.05 ± 4.74	22.01 ± 4.92	0.67

Data were expressed as number (percentage) or mean ± standard deviation.

Differences between circumcision and control groups were assessed by Chi-square test or *t*-test, as appropriate.

RMB: Renminbi; IELT: intravaginal ejaculatory latency time; IIEF-5: 5-item version of the International Index of Erectile Function.

**Table 2:** Outcomes of IELT and PRO measures for all subjects at baseline and each follow-up visit.

	Baseline (*N* = 1198)	Follow-up			
		3 months	6 months	9 months	12 months
		(*N* = 1122)	(*N* = 1076)	(*N* = 1038)	(*N* = 998)
**IELT, minutes**					
Circumcision group	1.58 ± 0.74^b,c,d,e^	1.44 ± 0.52^a,f^	1.86 ± 0.80^a,f^	2.08 ± 0.90^a,f^	2.11 ± 0.89^a,f^
Control group	1.55 ± 0.78	1.57 ± 0.75	1.61 ± 0.76	1.59 ± 0.78	1.58 ± 0.74

**PRO measures, scores**					
(A) Control over ejaculation					
Circumcision group	2.13 ± 0.93^b,c,d,e^	2.05 ± 0.83^a,f^	2.28 ± 1.04^a,f^	2.30 ± 1.02^a,f^	2.52 ± 1.12^a,f^
Control group	2.15 ± 0.85	2.16 ± 0.87	2.13 ± 0.82	2.20 ± 0.86	2.18 ± 0.83
(B) Satisfaction with sexual intercourse					
Circumcision group	2.24 ± 0.89^c,d,e^	2.25 ± 0.92	2.56 ± 0.94^a,f^	2.54 ± 0.92^a,f^	2.58 ± 0.98^a,f^
Control group	2.20 ± 0.84	2.21 ± 0.79	2.20 ± 0.85	2.18 ± 0.87	2.19 ± 0.85
(C) Severity of PE^*^					
Circumcision group	1.92 ± 0.69^c,d,e^	1.89 ± 0.65	1.54 ± 0.67^a,f^	1.49 ± 0.64^a,f^	1.50 ± 0.62^a,f^
Control group	1.94 ± 0.72	1.92 ± 0.70	1.90 ± 0.71	1.92 ± 0.71	1.91 ± 0.69
(D) Personal of distress					
Circumcision group	1.33 ± 0.80	1.32 ± 0.81	1.30 ± 0.79	1.28 ± 0.77	1.27 ± 0.75
Control group	1.39 ± 0.81	1.38 ± 0.83	1.39 ± 0.78	1.38 ± 0.80	1.39 ± 0.80
(F) Interpersonal difficulty					
Circumcision group	1.28 ± 0.74	1.24 ± 0.68	1.19 ± 0.67	1.30 ± 0.68	1.24 ± 0.65
Control group	1.26 ± 0.72	1.23 ± 0.71	1.22 ± 0.70	1.24 ± 0.72	1.21 ± 0.71

^a^Significant difference compared with baseline; ^b^significant difference compared with 3 months; ^c^significant difference compared with 6 months; ^d^significant difference compared with 9 months; ^e^significant difference compared with 12 months; ^f^significant difference compared with control group.

^*^Only included men with PE.

IELT: intravaginal ejaculatory latency time; PRO: Patient-Reported Outcome; PE: premature ejaculation.

**Table 3:** Outcomes of IIEF-5 for all subjects at baseline and each follow-up visit.

	Baseline (*N* = 1198)	Follow-up			
		3 months	6 months	9 months	12 months
		(*N* = 1122)	(*N* = 1076)	(*N* = 1038)	(*N* = 998)
(A) How do you rate your confidence about getting and keeping an erection?					
Circumcision group	4.29 ± 1.21	4.24 ± 1.19	4.24 ± 1.20	4.27 ± 1.16	4.33 ± 1.19
Control group	4.30 ± 1.14	4.31 ± 1.16	4.30 ± 1.19	4.34 ± 1.17	4.32 ± 1.16

(B) When you had erections with sexual stimulation, how often were your erections hard enough for penetration?					
Circumcision group	4.65 ± 1.43	4.52 ± 1.38	4.60 ± 1.32	4.59 ± 1.44	4.62 ± 1.37
Control group	4.59 ± 1.39	4.65 ± 1.40	4.63 ± 1.37	4.68 ± 1.52	4.60 ± 1.40

(C) During sexual intercourse, how often were you able to maintain your erection after you had penetrated your partner?					
Circumcision group	4.41 ± 1.76	4.45 ± 1.69	4.31 ± 1.82	4.28 ± 1.80	4.31 ± 1.74
Control group	4.39 ± 1.69	4.40 ± 1.72	4.34 ± 1.70	4.34 ± 1.65	4.33 ± 1.73

(D) During sexual intercourse, how difficult was it to maintain your erection to the completion of intercourse?					
Circumcision group	4.62 ± 1.45	4.56 ± 1.47	4.52 ± 1.39	4.58 ± 1.42	4.59 ± 1.39
Control group	4.64 ± 1.50	4.60 ± 1.51	4.60 ± 1.46	4.60 ± 1.48	4.61 ± 1.50

(E) When you attempted sexual intercourse, how often was it satisfactory for you?					
Circumcision group	4.08 ± 1.23^c,d,e^	4.25 ± 1.30^c,d,e^	4.70 ± 1.29^a,b,f^	4.72 ± 1.32^a,b,f^	4.46 ± 1.29^a,b,f^
Control group	4.09 ± 1.28	4.24 ± 1.27	4.32 ± 1.31	4.35 ± 1.28	4.30 ± 1.34

(F) Total scores					
Circumcision group	22.05 ± 4.74	22.03 ± 5.01	22.37 ± 4.90	22.44 ± 4.98	22.31 ± 5.21
Control group	22.01 ± 4.92	22.20 ± 4.92	22.19 ± 4.95	22.31 ± 5.14	22.16 ± 5.30

IIEF-5: 5-item version of the International Index of Erectile Function.

^a^Significant difference compared with baseline; ^b^significant difference compared with 3 months; ^c^significant difference compared with 6 months; ^d^significant difference compared with 9 months; ^e^significant difference compared with 12 months; ^f^significant difference compared with control group.

**Table 4:** Associations between circumcision, PE, and ED at 3-, 6-, 9-, and 12-month follow-up.

	PE		ED	
	Odds ratio	95% CI	Odds ratio	95% CI
3-month follow-up				
Control group	1	Ref	1	Ref
Circumcision group	1.13	1.05–1.42	NS	NS

6-month follow-up				
Control group	1	Ref	1	Ref
Circumcision group	0.82	0.53–0.98	NS	NS

9-month follow-up				
Control group	1	Ref	1	Ref
Circumcision group	0.56	0.42–0.79	NS	NS

12-month follow-up				
Control group	1	Ref	1	Ref
Circumcision group	0.37	0.24–0.57	NS	NS

PE: premature ejaculation; ED: erectile dysfunction; NS: not significant.

Associations between circumcision, PE, and ED were assessed by logistic regression.
